# Drying Performance of Fabrics on the Human Body

**DOI:** 10.3390/ma18112655

**Published:** 2025-06-05

**Authors:** Ivona Jerkovic, Agnes Psikuta, Sahar Ebrahimi, Joyce Baumann, Martin Camenzind, Simon Annaheim, René M. Rossi

**Affiliations:** Empa—Federal Laboratories for Material Science and Technology, Laboratory for Biomimetic Membranes and Textiles, Lerchenfeldstrasse 5, CH-9014 St. Gallen, Switzerland; ivona-jerkovich@outlook.com (I.J.); martin.camenzind@empa.ch (M.C.); simon.annaheim@empa.ch (S.A.); rene.rossi@empa.ch (R.M.R.)

**Keywords:** textile materials, wetting, drying, drying methods, drying time, drying rate

## Abstract

When developing fabrics for applications in which evaporative cooling and drying play an important role, e.g., sports or occupational applications, the drying performance of fabrics is commonly determined using fast and easy-to-perform benchmark methods. The measurement conditions in these methods, however, differ significantly from the drying conditions on the human body surface, where drying is obstructed on one side of the fabric through contact with the skin and at the same time enhanced due to contact with the heated surface (skin). The aims of this study were to understand and quantify the fabric drying process at the skin interface considering these real-use effects based on tests applying two-sided drying, one-sided drying, one-sided drying on a heated surface, and one-sided drying on a heated surface in the stretched state, and to relate these to existing standard methods. The findings showed that contact with a solid heated surface such as the skin and the stretched state of the fabric both make a significant contribution (*p* < 0.05) to the drying rate compared to two-sided drying in standard climatic conditions. The corresponding drying rates observed for a range of typical fabrics used in leisure and sports as a first layer next to the skin were found to be 1.6 (±0.2), 1.1 (±0.2), 7.9 (±2.1), and 10.6 (±0.8) g/m^2^ min for two-sided drying, one-sided drying, one-sided drying on a heated surface, and one-sided drying on a heated surface in the stretched state, respectively. These findings are of great importance for human thermal modelling, including clothing models, where the drying process significantly contributes to the heat and mass transfer in the skin–clothing–environment system.

## 1. Introduction

**Figure 1:**
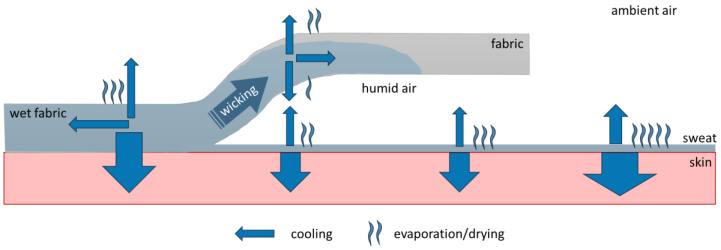
Overview of the evaporative cooling process between skin, clothing, and environment.

Physical activity and/or exposure to warm or hot conditions induces sweating. Some of the sweat is absorbed by the clothing fabric and evaporates from there; evaporation of sweat from the fabric rather than from the skin may lead to a lesser body cooling effect, due to the distance between the fabric and the skin, and may also lead to a delay in cooling due to the difference in evaporation rates from the bare skin and the structure of the fabric [1] (Figure 1). Finally, the sweat moisture picked up by the garment may be accumulated in the clothing if the sweat production rate is greater than the evaporation rate over a period of time, and this may lead to continuous cooling of the body even after the secretion of sweat has ceased and cooling is no longer needed from the point of view of thermoregulation. This effect is commonly called post-exercise chill and is typically to be avoided, especially when exercising in cold conditions. Besides issues related to cooling, the accumulated moisture may lead to so-called wet discomfort due to the dampness and clinginess of clothing [2,3,4,5,6,7,8,9].

When developing fabrics for applications where evaporative cooling and drying play an important role, e.g., sports or occupational applications, the drying performance is typically compared between successive prototypes in the development process, as well as products on the market, using fast and easy-to-perform benchmark methods. There are several standardised benchmark methods that can determine the drying times and drying rates of wetted textile fabrics (Table 1). These methods differ in terms of the fabric orientation (vertical or horizontal), the amount of moisture delivered superficially to the fabric (for example, 0.08–5 mL or fully wetted), and the thermal surroundings of the sample (ambient temperature and relative humidity of 20–35 °C and 40–65%, respectively, and/or contact with a heated surface at 35–37 °C) (e.g., ISO13029 [10], ISO 17617 [11], AATCC 195-2011 [12], AATCC 199-2011 [13], AATCC 200-2017 [14], and AATCC 201-2014 [15]). This means that they are not equivalent; instead, they are suitable for different purposes and reveal fabric drying performance under various real-use conditions. In addition to these standard methods, scientists have provided various altered methods suited to a particular research purpose, for example, by adding an increased air speed that can accelerate the drying process [16], an increased amount of moisture compared to standard conditions for the evaluation of the drying rate of knitted fabrics [17], or the use of a smaller guarded hotplate and a reduced amount of moisture [18].

The scientific literature offers a rich database of drying rates for fabrics measured under laboratory conditions according to standard and modified standard gravimetric methods (particularly ISO 17617, methods A1 and A2 [11]). The basic principle of such measurements is to apply a certain amount of water to a swatch of a given fabric and to let it dry, hanging freely or lying on a surface, under standard environmental conditions while the sample mass is monitored over time. Such an approach allows for a comparison of drying rates between materials and is relatively simple to perform and affordable (as only a precision balance and a conditioned laboratory space are required in many cases, or moderately expensive automatised benchmark testers). These measurement conditions, however, differ significantly from the drying conditions on the human body surface, where drying is obstructed on one side of the fabric through contact with the skin and at the same time enhanced due to contact with the heated surface (skin). The elevated temperature of the fabric and the adjacent air layer increase the evaporation potential from the fabric, since warmer air can accommodate a higher water vapour partial pressure and hence create a greater water vapour partial pressure gradient, which is a driving force for evaporation and water vapour diffusion.

Secondly, the amount of moisture accumulated in the fabric will influence its drying performance, due to potential changes in local evaporation when a larger area is wet. The moisture initially evaporates from the fabric to the air space within the fabric structure and into the thin adjacent air layer at the fabric surface. Finally, it is evacuated away from the fabric by a diffusion process driven by the water vapour partial pressure gradient between the evaporation space and the surroundings. In most standard methods, the amount of test water put onto the fabric is small (0.08–0.3 mL in ISO17617 [11] and 5 mL in ISO 13029 [10] as a spot wetting amount), and the drying process progresses simultaneously with the wicking process. Since the evaporation rate depends on the wetted area, the drying rate measured by such methods will include artefacts of the wicking speed and radius, which may obscure a comparison of the drying and cooling performance of the samples even if they are measured by the same method.

The results from the benchmark methods frequently raise the questions of whether the fabric’s improved drying (or cooling) performance makes a significant difference for the wearer in critical situations and whether it improves thermal comfort. These issues can be investigated through human wear trials or by using adaptive manikins. Both approaches demand substantial effort in terms of garment preparation (as opposed to fabric swatches), determining an exposure scenario in which the differences between the samples of interest can be revealed, both involving substantial cost and time investments. In contrast, simulation tools focusing on human thermo-physiology, thermal sensation, and clothing offer a fast and more versatile alternative to resolving multiple use cases in sports and occupational situations with relatively low effort. To obtain a reliable simulation of the human response using such tools, an adequate definition of the clothing needs to be provided, including its local thermal and evaporative properties and the changes in these quantities during use (i.e., a comprehensive clothing model is needed). To predict the correct magnitude and duration of evaporative cooling, the clothing model used should be able to properly reflect the moisture accumulation and evaporation from the clothing layers. However, none of the existing models addresses this issue in detail; instead, they focus either on the microscopic wetting and drying processes in the porous structure of the fabric (without considering the context of other clothing, air layers, and physical processes occurring during wear) (e.g., Farnworth [19] and Gibson [20]) or on the general mass flow through the entire clothing system without accounting for the local accumulation of moisture and its potential thermal effect (e.g., Joshi [21,22,23] and Lotens [24,25,26,27]). This gap is mainly related to a lack of understanding of how to use the knowledge from the existing benchmark methods under distinct test conditions (Table 1) and how to implement it in a comprehensive clothing model.

The aim of this study was to understand and quantify the fabric drying process at the skin interface, considering the obstruction of evaporation by the skin and drying enhancement by the heated skin surface. This was investigated using a gravimetric method based on fabric samples suspended in the air or in contact with a non-heated or heated surface. The outcome of this study should provide a perspective on making choices and understanding existing versatile benchmark methods for applications where drying is a critical performance landmark. Secondly, it will help in implementing this knowledge in clothing modelling and models of human thermoregulation and comfort, where quantification of drying time and evaporative cooling is essential for accurate prediction of human comfort.

**Table 1:** Overview of drying test methods found in existing standards and the literature, with influential parameters of the evaporation process.

Test Method	Scope	Lab Conditions	Amount of Moisture	Sample Size	Sample Orientation	Contact Surface
Dynamic drying rate using sweating-guarded hotplate, ISO 13029:2012 [10]	All fabric types	35 °C, 40%, 1 m/s	5 mL	30 × 30 cm	Horizontal	Plate at 35 °C
Moisture drying rate in standard air conditions, ISO 17617:2014 [11]	All fabric types	20 °C, 65%, 0.1 m/s	0.3 mL 0.08 mL	20 × 20 cm 10 × 10 cm	Vertical	None
			0.1 mL	Ø 8.5 cm	Horizontal	Petri dish
Liquid moisture management test, AATCC 195-2011 [12]	Knit, woven, and non-woven fabrics	21 °C, 65%, no control of air speed	0.22 g	8 × 8 cm	Horizontal	None
Drying time using moisture analyser method, AATCC 199-2011 [13]	Absorbent materials	37 °C, 65%, no control of air speed	Variable, sample fully wet	Ø 7 cm	Vertical	None
Drying rate of textiles with absorbent capacity using air flow method, AATCC 200-2017 [14]	Absorbent materials	21 °C, 65%, 2.5 m/s	0.1 mL ± 0.003 mL added in steps	15 × 15 cm	Horizontal	None (specimen mounted in ring)
Drying rate of fabrics using heated plate method, AATCC 201-2014 [15]	All fabric types	21 °C, 65%, 1.5 m/s	0.2 mL	15 × 15 cm	Horizontal	Plate at 37 °C
Drying time of fabrics, Chakroun et al. [17]	Knitted fabrics	20 °C, 65%, 0.1 m/s	20 mg	Circular specimen, 100 cm^2^	Horizontal	Petri dish
Isothermal drying rate at constant air speed, Heinisch et al. [16]	Woven fabrics	21 °C; 50%; 2, 3, or 4 m/s	Variable, sample fully wet	11 × 8 cm	Horizontal	Plate
Bimodal fabric drying kinetics, Hussan et al. [18]	Knitted fabrics	35 °C, 40%, 1 m/s	1.4 mL	30 × 30 cm	Horizontal	Plate at 35 °C
Drying rate of fabrics for sport clothing, Fangueiro et al. [28]	All fabric types	22 and 33 °C, 65%, 0.1 m/s	30% of dry fabric weight	20 × 20 cm	Horizontal	Plate
Drying rate of fabrics, Cay et al. [29]	All fabric types	20 and 35 °C; 27, 53, and 65%; 0.1 m/s	1.0 g	Circular specimen, 50 cm^2^	Horizontal	Plate
Drying behaviour of fabrics and garments [30,31]	All fabric types	20 °C, 65%, 0.1 m/s	Variable, sample fully wet	4 × 4 cm	Vertical	None

## 2. Materials and Methods

### 2.1. Methodological Approach

In order to study the correlation between the drying performance of fabrics measured with existing standard benchmark methods and under real-use conditions, the following four methods were implemented and compared:

(1) A standard method using droplet wetting and a free hanging sample, according to ISO 17617 [11], method A2 (two-sided drying of a rather small wetted area);
(2) A method like (1) but with a fully wetted sample (two-sided drying);
(3) A method like (1) but with a fully wetted sample wrapped around a plastic cylinder to obstruct evaporation from the back side of the fabric facing the cylinder (one-sided drying);
(4) A method using a vertical heated cylinder wrapped in a fully wetted sample under ambient conditions, as in (1) (one-sided drying on heated surface).

This series of methods allowed for a systematic analysis of the fabric drying behaviour at the skin compared to a lab standard method and a systematic estimation of the individual contributions of different exposure conditions by adding one additional effect at the time, i.e., full sample wetting in (2), one-sided evaporation obstruction in (3), and a heated surface with increased evaporation potential in (4). All methods were compared based on a pool of samples representing typical casual and leisure sport fabrics with different fibre contents, structures, and stretch levels (20%). Secondly, groups of fabrics with either the same fibre content or structure or stretched state were defined for additional systematic comparisons.

### 2.2. Materials

In this study, fabric samples were selected according to the most influential parameters affecting the fabric drying performance, such as fibre content, which influences hydrophilicity and hygroscopicity, and the fabric structure and stretch level, which influence the void structure and volume available for water accumulation and subsequent drying.

The fibre types included in this study ranged from natural fibres, such as cotton, wool, and regenerated fibres (lyocell and modal), to conventional synthetic fibres, such as polyester and polyamide. Furthermore, some fabrics selected for this work had a certain percentage of elastane in their composition, as indicated in Table 2. This range of fibre types represented a wide range of drying performances, as reported in previous studies [32], including samples with fast (e.g., PES-P and PA-I), medium, and slow (e.g., MM-J and HWO-J) drying rates. Secondly, different fabric structures were chosen within the same fibre content samples (i.e., PES-I, PES-J, PES-L1, and PES-P in the polyester group and CO-S, CO-I, and CO-J in the cotton group) in order to cover a variety of casual and leisure sport fabrics and at the same time provide some insights into the effect of structure on drying behaviour. Finally, we measured a group of single jersey fabrics in a stretched state of 20% elongation under the same conditions as the unstretched fabrics (i.e., PES-J, CO-J, HWO-J, WO-J, and MM-J) to study the effect on the drying rate, given that many applications of single jersey fabrics require their use in a stretched state.

The specific properties of each fabric, including microscopic images of their surfaces from both sides (KEYENCE VHX1000, Mechelen, Belgium; magnification: 100×), are given in Table 2. The physical properties of the selected fabrics, such as their mass per unit area and thickness, were determined according to ISO 3801 [33] and ISO 5084 [34], respectively. Thickness tests were conducted using a thickness tester (D-2000-T thickness gauge, Hans Schmidt Co GmbH, Waldkraiburg, Germany), air permeability tests were conducted according to ISO 9237 [35] using an air permeability tester that was built in-house (with a test surface area of 20 cm^2^ and a pressure drop of 100 Pa), and finally contact angles were measured using a benchtop Drop Shape Analyser (Krüss GmbH, Hamburg, Germany). Thickness and air permeability tests were also performed for single jersey fabrics in the stretched state (20% in the course direction, as for the drying test) using a dedicated sample holder (with a test surface area of 100 cm^2^).

The relative porosity, *p*, was calculated using the following equation [36]:

(1) $$P=\left(1-\frac{m}{\rho \times d}\right)\times 100\%$$

where *m* is the fabric mass per unit area (g·m^−2^), *ρ* is the fibre density (g·m^−3^), and *d* is the fabric thickness (m).

### 2.3. Sample Preparation

All fabrics were washed once, according to ISO 6330 [37] (at a washing temperature of 40 °C, using 100% polyester ballast, 20 g of reference non-phosphate powder detergent, a drum rotation of 800 rpm, and a washing duration of 75 min). The fabric samples were cut and sewn into tunnels for tests on cylinders. The samples to be tested in the stretched state were prepared in the form of a tunnel with an anticipated stretch level of 20% (where the sample circumference was 20% smaller than the cylinder circumference). Following this, all samples were conditioned at an ambient temperature of 20 ± 2 °C and a relative humidity of 65 ± 2% for 24 h before undertaking the tests, including fabric characterisation tests, as listed in Table 2. Three samples of each fabric were tested using all protocols, except for the method following ISO 17617 [11], where five samples of each fabric were tested.

### 2.4. Protocols

All tests were performed in a climatic chamber set to test conditions of 20 ± 1 °C for ambient temperature and 65 ± 5% for relative humidity. The acclimatised samples were wetted for 1 h prior to each drying test (apart from the samples wetted with a droplet) through immersion in deionised water. Samples in the stretched state were measured only on the heated cylinder. A precision balance (Mettler AE240-S, Mettler-Toledo, Switzerland; precision: 0.001 g, accuracy: ± 0.05 g) connected to a computer with automatic weight monitoring software, a stand with a hook, and an enclosure to minimise the air speed in the proximity of the sample to 0.1 m/s were used in all the experiments apart from the one on the heated cylinder.

#### 2.4.1. Gravimetric Method with Two-Sided Drying (Droplet Wetting)

**Figure 2:**
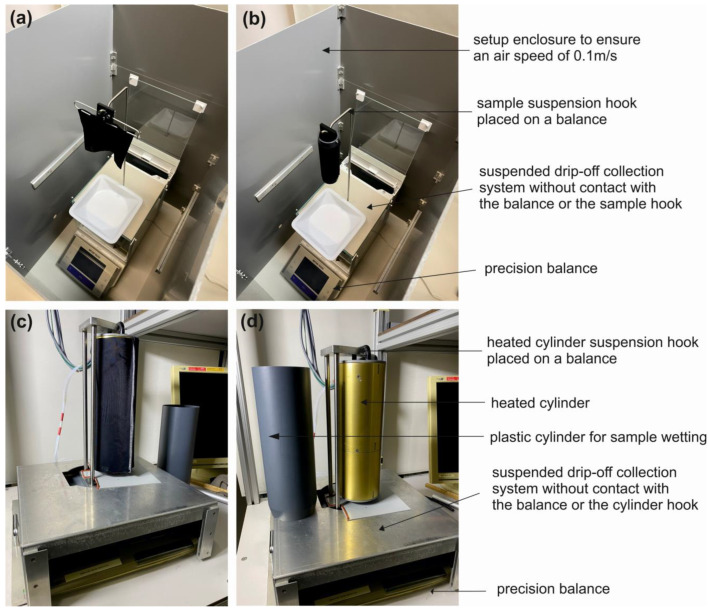
Measurement setups for (**a**) the gravimetric method with two-sided drying for both droplet and fully wetted samples, (**b**) the gravimetric method with one-sided drying, and (**c**) the gravimetric method with one-sided drying on a heated surface. (**d**) The heated cylinder setup was suspended on a precision balance, and the plastic cylinders used for wetting the samples were of identical size.

The drying time was measured according to ISO 17617 [11], using method A2 for square fabric samples (10 × 10 cm) wetted with 0.08 mL of water at their centre using a pipette (Figure 2a). The mass loss of a hanging sample was observed for up to 90 min. The validation test was performed on all specimens prior to the test except for four fabrics (PES-P, CO-I, PA-I, and MM-J, as shown in Table 2) due to insufficient absorbency (where the absorption time was greater than 60 s). The drying time was recorded at 95% of applied water loss.

#### 2.4.2. Gravimetric Method with Two-Sided Drying

Measurement of the drying time was performed according to ISO 17617 [11] with method A2, except for the sample that was fully wetted. The square fabric samples (10 × 10 cm) were reinforced with thin needles along the sample edges to prevent sample rolling, which would have caused accumulation of surface water and obstruction of drying, and they were subsequently immersed in deionised water for wetting. Each wet sample was hung on a stand with a hook on a precision balance, and the mass loss was continuously recorded. A container to collect the dripping water was placed below the sample, without contact with the balance. The mass change for each specimen was automatically recorded at intervals of 20 s until 95% of the taken-up water was evaporated (Figure 2a).

#### 2.4.3. Gravimetric Method with One-Sided Drying

Gravimetric measurements of the drying time were made using a small hollow plastic cylinder (with a diameter of 3.18 cm and a height of 13 cm) to allow for evaporation (drying) only on one side of the fabric. A square fabric sample (10 × 10 cm, including 2 × 0.5 cm of seam allowance) was sewn into a tunnel and put onto the hollow cylinder and then wetted through immersion in deionised water. The wet sample on the hollow cylinder was then hung on the stand with a hook placed on a precision balance, and the mass loss was continuously recorded at intervals of 20 s until 95% of the taken-up water was evaporated. A container for the dripping water was placed below the sample, as described earlier (Figure 2b).

#### 2.4.4. Gravimetric Method with One-Sided Drying on a Heated Surface

A heated cylinder (with a diameter of 10.2 cm and a height of 28 cm) was used to carry out a one-sided drying test on a heated surface (Figure 2d). A brass cylinder was heated electrically with four heating foils on the inner side of the brass cylinder shell. Four Pt100 sensors built onto the cylinder surface were used to measure the surface temperature with a calibrated accuracy of 0.05 °C. The drying test was run with the cylinder set at a constant surface temperature of 35 °C, and variable heating power was needed to maintain this temperature. The entire cylinder was suspended on a balance (Mettler SM 1220, Mettler-Toledo, Switzerland; precision: 0.1 g), allowing for separation of the sample mass and the dripped-off water mass.

The fabric sample (with dimensions of 28 × 31.4 cm, including 2 × 0.5 cm seam allowance) was sewn along one side (28 cm side) to form a tunnel and was then put on a hollow plastic cylinder of the same size as the heated cylinder and immersed in deionised water. This intermediate step allowed for wetting of both stretched and unstretched samples, since the heated cylinder itself could not be immersed in water. By aligning the bases of the plastic cylinder with the wetted sample and the bare heated cylinder, the sample could be gently slid down from the plastic cylinder onto the heated cylinder with minimal accidental water loss from the wetted sample. Finally, the heated cylinder with the wetted sample was placed on the balance using a suspension system, and a plastic tray for the dripping water was placed underneath (Figure 2c). The mass change of each sample was recorded every minute for 150 min, which was defined as the testing time and was sufficient for the drying process to be completed (as confirmed in preliminary tests). Measurements were considered complete when 95% of the taken-up water was evaporated.

### 2.5. Calculations and Statistical Analysis

The maximum water content (*MWC* (g)) reached in the methods applied in this study was calculated using the following formula:

(2) $$MWC=\frac{m_{wet}-m_{dry,20\;{}^{\circ}C,65\%}}{A}$$

where *m_wet_* (g) is the wet mass of the sample at the beginning of the drying test, *m_dry_*_,20 °C,65%,_ (g) is the dry mass of the sample under test conditions measured prior to the wetting process, and A is the surface area of the sample (m^2^). In the test using a heated cylinder, the value of *m_dry_* was determined at the end of the test when the sample was fully dry, for a cylinder temperature of about 35 °C. The drying rate (*DR* (g/m^2^ min)) of fabric samples was calculated using the following formula:

(3) $$DR=\frac{m_{wet}-m_{95\%dry}}{t\times A}$$

where *m_wet_* (g) is the wet mass of the sample at the beginning of the drying test, *m_95% dry_* (g) is the mass of the sample at time *t*, *t* (min) is the time at which 95% of the maximum water content had evaporated, and *A* (m^2^) is the surface area of the sample. Mean values and standard deviations for the maximum water contents, drying times, and drying rates were calculated based on the numbers of repetitions for all methods.

The differences in the outcomes under the various effects were investigated by performing an independent *t*-test (two-tailed distribution assuming equal variance) in Microsoft Excel (Microsoft Office 365, Microsoft Corporation, Redmont, WA, USA) with the significance threshold set to *p* < 0.05. A separate bivariate correlation analysis (Pearson) was conducted to investigate how the maximum water content, drying time, and drying rate were related to the fabric properties, such as thickness, mass, and air permeability, for the methodologies and groups of materials considered in this study. A linear regression analysis was applied to quantify how the fabrics (both overall and the individual materials) were related to the drying characteristics (maximum water content, drying time, and drying rate), as measured by the different methodologies. The differences between the regression lines (slopes) were tested with an independent *t*-test, corrected for multiple testing (Bonferroni), where applicable. For the bivariate correlation analysis, IBM SPSS Statistics software (version: 28.0.1.1) was used (IBM, Armonk, NY, USA).

## 3. Results

**Figure 3:**
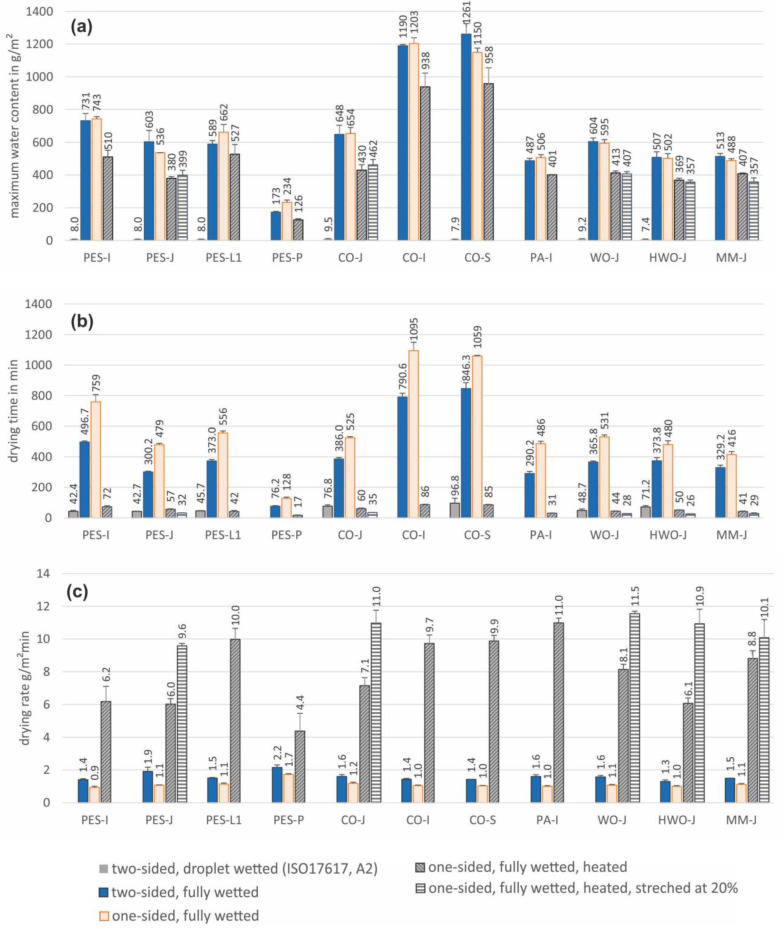
(**a**) Maximum water content, (**b**) drying time, and (**c**) drying rate for wetted fabric samples evaluated by four methods: ISO17617 [11] (droplet wetting), two-sided drying of a flat sample, one-sided drying on a cylinder, and one-sided drying method on a heated cylinder, in the unstretched and stretched states.

**Figure 4:**
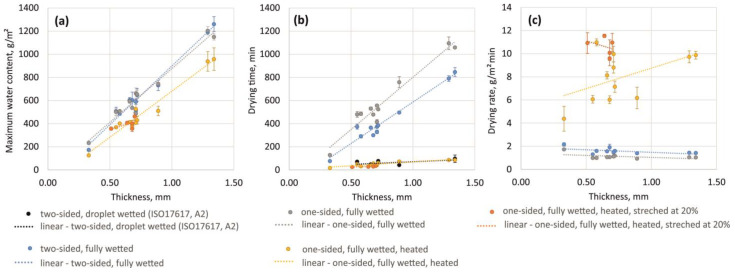
Correlations between (**a**) maximum water content, (**b**) drying time, and (**c**) drying rate in relation to fabric thickness for all fabrics and all methods.

The results for the maximum water contents, expressed in g/m^2^, for the fully wetted fabric samples from the three methods (two-sided drying, one-sided drying, and one-sided drying on a heated surface) are presented in Figure 3a. The fourth method, based on ISO17617:2014 (two-sided drying with droplet wetting), used a defined amount of moisture (0.08 mL), resulting in a constant water content of 8 g/m^2^, and the results are also presented in Figure 3a. The drying times for all fabrics obtained using all four methods, expressed in minutes, are presented in Figure 3b. Data for the method according to ISO17617 [11] are not provided for the four samples (PES-P, CO-I, PA-I, and MM-J) that did not pass the validation pre-test according to this standard. The drying rates, expressed in g/m^2^ min, for fully wetted fabric samples under the three methods (two-sided drying, one-sided drying, and one-sided drying on a heated surface) are presented in Figure 3c. The drying rate for the method according to ISO17617 [11] is not given, since the wetted area of the sample was not known (wetting was applied with a droplet in the sample centre, and the wetted area depended on the wicking properties of the sample, meaning that it was different for each sample and changed over time). The values of all three parameters measured in the stretched state are provided for only single jersey samples (PES-J, CO-J, WO-J, HWO-J, and MM-J) (Figure 3a,b). Table 3 lists the significance levels at *p* < 0.05 of the differences between subsequent methods corresponding to the added effects of full wetting, obstruction of evaporation at the back of the fabric, use of a heated surface, and stretch level, for all evaluated fabrics. Correlations between maximum water content, drying time and drying rate, and thickness for all of the investigated methods, including correlation lines, are presented in Figure 4. The specific values of the linear correlations (slope, intercept, R^2^, and corresponding standard errors) for all fabrics and correlations between individual groups of fabrics (CO-based, PES-based, and jersey fabrics) and the methods used are summarised in Table 4 and Table 5.

## 4. Discussion

A systematic comparison of the four different methods used to investigate the drying behaviour of a variety of fabrics showed a significant difference between the drying time and drying rate results between all methods (see Table 3, values marked with *). The drying time according to the ISO17617 [11] A2 method based on droplet wetting (0.08 mL) and the drying time for a fully wetted sample of the same size and under the same environmental conditions were expected to be different (Figure 3b), mainly because of the great difference in the moisture content of the samples in these tests (on average, 86-fold, with a range between 65- and 160-fold more moisture for the fully wetted samples; Figure 3a). The drying rate would have been a better parameter for comparison between these two methods, since it is independent of the initial moisture content of the sample; however, it was not possible to obtain the drying rate for the ISO17617 [11] A2 method, as the wetted area changed dynamically over the initial testing time due to the wicking process of the moisture applied to the middle of the sample. This is a major drawback of methods based on droplet wetting (e.g., ISO17617 [11] and ISO13029 [10]), which recommend different amounts of moisture and sample sizes, meaning that the drying times cannot be compared. In addition, the determination of drying time based on the droplet wetting approach is burdened with the artefact of the wicking properties of the sample, which interfere with the drying process (the greater the wetted area, the faster the drying process) [38] and hence make the comparison between samples with distinct wicking properties questionable, even when using the same method for drying evaluation. The absorbency test prior to the actual drying test offered in ISO17617 is an attempt to mitigate this issue by excluding highly hydrophobic samples (in our study, PES-P, CO-I, PA-I, and MM-J were excluded, which, as expected, presented the smallest contact angle property amongst all fabrics in this study; see Table 2). Droplet wetting does not reflect realistic conditions for the wetting of fabrics during perspiration of the human body. The sweating pattern on the human body is rather areal, involving millions of sweat glands distributed over the skin and seldom showing great contrasts in sweat rates at different points nearby [39,40,41,42,43].

Another effect that is common in the realistic drying of fabrics on the human body is obstruction of the evaporation (and hence drying) on the inner side of the garment, either due to direct contact with the skin or to the increased relative humidity in the air gap under the garment caused by sweat evaporation from the skin. We observed that obstructing evaporation on the inner side of the fabric by using a plastic cylinder and wrapping the fabric sample tightly around it significantly increased the drying time and decreased the drying rate for all tested samples (Table 3, Figure 3b,c). The presence of the cylinder, however, did not significantly affect the maximum water content of the sample (except for CO-S and PES-P, which showed insignificant differences), and thus the drying times obtained by both methods could be directly compared (for the same sample size, moisture content, and environmental conditions). Obstructing evaporation at the back of the sample prolonged its drying time by 52–304 min (on average, 170 min for all samples tested) and decreased the drying rate by 0.30–0.85 g/m^2^ min (on average, 0.46 g/m^2^ min for all samples tested). Amongst methods available for fabric drying evaluation (as shown in Table 1), both methods, i.e., with and without contact with a solid surface causing evaporation obstruction, are represented. In ISO17617 [11], both methods are equally recommended within the same standard. As long as samples are benchmarked with the same method, a comparison between the results is possible (although there are limitations due to the presence of the wicking effect mentioned earlier), but the prediction of realistic drying in use conditions is not possible. All methods provided a similar classification of the variety of fabrics selected for this study with regard to drying time (the only parameter available for all methods), but only for samples with large drying-time differences. For example, all methods identified PES-P as the fastest-drying fabric and CO-I and CO-S as drying at the slowest rates (Figure 3b,c). For fabrics with smaller differences in drying time, the classification varied between methods. For example, sample HWO-J was in the middle range in terms of drying time and was classified as the fifth-, seventh-, fourth-, and sixth-fastest drying fabric by the two-sided drying method with droplet wetting, two-sided drying, one-sided drying, and one-sided drying on a heated surface, respectively.

Under more realistic conditions, an additional effect of enhanced evaporation potential (and hence higher drying rate) can be induced by heating fabric and the adjacent air layer by warm skin. This was simulated in our tests with a heated cylinder, and the results were compared to the case of the unheated cylinder (Figure 3). The drying rate was significantly higher with the heated cylinder for all samples (Table 3), with an increase of between 2.5 and 11.1 times the drying rate for the unheated cylinder (with an average increase of 7.4 times for all samples tested). The maximum water content in this test was found to be lower than in other tests with fully wetted samples, due to the water loss during transfer of the sample from the plastic cylinder used for wetting of the samples to the heated cylinder used to measure the drying rate. The heated cylinder could not be directly immersed to wet the sample because it was not watertight when submerged in water. The difference in maximum water content between the wetted samples on the plastic cylinder compared to the samples on the heated cylinder was 27% lower on average (between 17% and 46% for all samples), which shortened the drying time for that test. The transfer of the wetted sample onto the heated cylinder also increased variability in repetitive measurements between both methods (4% and 6% for the one-sided drying and the one-sided drying on a heated surface methods, respectively). Nonetheless, the mass loss observed for all samples was directly proportional to the time in all methods (i.e., a linear correlation), which makes the comparison of drying rates between methods a valid approach despite the reduced initial moisture content in the heated cylinder method.

The single jersey fabric samples were also examined in a stretched state (20%) to observe their drying properties under realistic use conditions, but no significant difference in maximum water content was found between the stretched and unstretched states (Table 3, Figure 3a). However, the drying time for all jersey samples was significantly shorter, and the drying rate was significantly higher in the stretched state (with the exception of the MM-J sample, where the difference in drying rate was not significant). This may be related to the increased space for evaporation and diffusion within the fabric structure, created by enlarged pores between yarns, which also resulted in increased air permeability of the stretched samples compared to the relaxed state [44,45] (see Table 2).

Finally, correlation analyses were conducted for the drying performance of fabrics and their physical characteristics. All three parameters relevant to the fabric drying performance (maximum water content, drying time, and drying rate) were significantly linearly correlated with fabric thickness, mass, and air permeability, and the best correlation was observed for fabric thickness for all methods and parameters tested (Table 4). Next, the individual groups of fabrics defined in this study (CO-based, PES-based, and single jersey fabrics) were also analysed for correlations between drying performance and fabric thickness using all methods. In all cases, the individual correlation slopes did not significantly differ from those observed for the remaining fabrics (*p* < 0.05). This indicates that the fabric thickness affects drying performance much more than the fabric material (CO-based, PES-based, and single jersey fabrics). Similar findings have also been reported in other studies [30,46,47,48,49,50].

The correlation equations obtained for the various methods (Table 5) illustrate the differences to be expected when evaluating fabric drying performance. The maximum water content was found to be the same for the fully wetted fabrics in the two-sided and one-sided drying methods. This was as expected, since the wetting process was similar for both methods and the wetting time was long enough to overcome the wetting obstruction for the one-sided approach (plastic cylinder, Figure 4a). The slope for the maximum water content in the heated cylinder method was, however, significantly smaller than for the other two methods, due to the water loss that occurred during sample transfer between the two cylinders, which represents a limitation of this test method. Furthermore, this water loss seemed to be greater for thicker fabrics, as the slope for the heated cylinder method was smaller than those for the two- and one-sided drying methods (Figure 4a).

The correlation equations for drying time can only be directly compared for methods based on fully wetted fabrics (three methods). In the approach based on ISO17617 [11], the initial water content was much smaller and hence gave the shortest drying time for all methods. When comparing the drying-time equations for the two- and one-sided drying methods, the effect of evaporation (and hence drying) obstruction at the back of the fabric became apparent and quantifiable. The drying time for the one-sided drying method (plastic cylinder) increased on average by 45% compared to two-sided drying (between 25% and 69% for all samples tested, Figure 3b). In general terms, the slope for the increase in drying time with fabric thickness changed from 719 to 907 min/mm (Table 5); however, this significantly decreased from 907 to 66 min/mm when heating of the cylinder surface was added (heated cylinder method). Since the initial water content in this test was lower than in the other two methods, as discussed previously, the drying time was biased. When corrected for this initial water loss, the slope of the drying time with fabric thickness was approximately 80 min/mm (compared to 66 min/mm resulting from the original measurements). These differences in the drying time resulted in corresponding differences in the drying-rate correlation equations for the two- and one-sided drying methods offset by 0.5 g/m^2^ min (drying obstruction on the back side of the fabric), by 6.8 g/m^2^ min for one-sided drying on a heated cylinder compared to an unheated cylinder (increased evaporation potential), and by 2.7 g/m^2^ min for one-sided drying on a heated cylinder in the stretched state compared to relaxed states. The correlation of the drying rate with fabric thickness was found to be insignificant for all methods and all fabrics (Table 5, Figure 4c), a finding that is consistent with those of other studies that the drying rate is independent of the fibre content or fabric thickness [46,47,48]. Some significant correlations were observed for specific fibre groups (Table 5), but due to the limited data for the fibre-specific correlation analysis (3 specimens for CO and 4 specimens for PES), these results should be interpreted with caution.

The findings of this study are of great importance for human thermal modelling, including clothing models, where the drying process significantly contributes to heat and mass transfer in the skin–clothing–environment system. Quantification of the differences between the different drying scenarios that are typical for clothing systems (and associated measurement methods) can provide a basis for an accurate account of evaporative heat loss and drying in a mathematical clothing modelling approach. The clothing model with integrated general mass flow through the entire clothing system (e.g., Joshi [21,22,23] and Lotens [24,25,26,27]) can be extended with algorithms predicting local accumulation of moisture and the drying rate, including its potential effect on evaporative cooling. As a further step, an investigation of the dependence of the drying rate on air humidity and the temperatures of air and skin could further enhance the prediction accuracy of such clothing drying algorithms.

## 5. Conclusions

This study provides insights into and quantification of fabric drying rates in different scenarios related to the real use of clothing, unlike the majority of presently used standard benchmark methods. Our findings show that contact with a solid surface, contact with a solid heated surface (such as skin), and the stretched state of fabric all make significant contributions to the drying rate as compared with two-sided drying in standard climatic conditions. The drying rates observed across a range of typical fabrics used in leisure and sports as a first layer next to the skin were found to be 1.6 (±0.2), 1.1 (±0.2), 7.9 (±2.1), and 10.6 (±0.8) g/m^2^ min for two-sided drying, one-sided drying, one-sided drying on a heated surface, and one-sided drying on a heated surface in the stretched state, respectively.

The findings of this study are of great importance for human thermal modelling, including clothing models, where the drying process significantly contributes to the heat and mass transfer in the skin–clothing–environment system. They also highlight some potential issues and limitations of the standard benchmark methods that are currently used to determine the drying performance of fabrics and when interpreting the metrics of these methods in the context of their effect on the thermal state and comfort of humans.

## Figures and Tables

**Table 2:** Characteristics of fabric samples used in this study, including fibre composition, structure, mass, thickness, air permeability (AP), fabric density, relative porosity, and contact angle (fabric thickness and air permeability were also determined for single jersey fabrics in the stretched state at 20%). Scale bar length in microscopic pictures is equal to 1 mm.

Fabric Code	Composition	Structure	Front Side	Back Side	Mass per Unit Area (g/m^2^)	Thickness/Thickness Stretched (mm)	AP/AP Stretched (mm/s)	Fabric Density (g·cm^−3^)	Relative Porosity (%)	Contact Angle (°)
PES-I	100% PES	Interlock	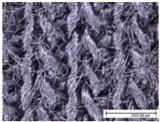	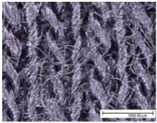	232	0.89 ± 0.01	1028 ± 120	1.38	81%	116 ± 6
PES-J	98% PES 2% EA	Single Jersey	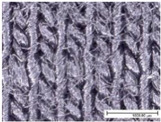	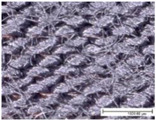	227	0.68 ± 0.01/ 0.68 ± 0.01	428 ± 18/ 908 ± 42	1.38	76%	121 ± 8
PES-L1	100% PES	Bird’s eye	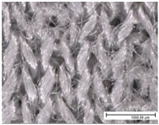	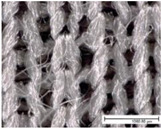	149	0.71 ± 0.01	1885 ± 29	1.38	85%	112 ± 12
PES-P	81% PES 19% EA	Warp knitted mesh (power mesh)	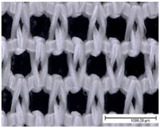	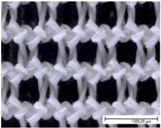	109	0.33 ±0.00	>4175	1.33	75%	107 ± 6
CO-J	98% CO 2% EA	Single jersey	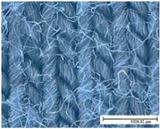	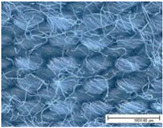	200	0.72 ±0.01/ 0.70 ± 0.01	163 ± 16/ 614 ± 12	1.53	82%	/ Highly hydrophilic
CO-I	100% CO	Interlock	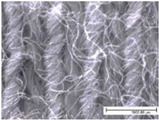	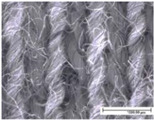	252	1.29 ± 0.01	506 ± 53	1.54	87%	97 ± 4
CO-S	95% CO 5% EA	French terry (sweatshirt)	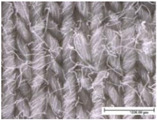	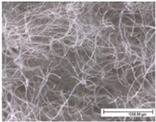	245	1.34 ± 0.01	262 ± 33	1.52	88%	/ Highly hydrophilic
PA-I	100% PA	Interlock	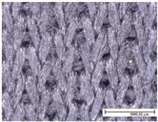	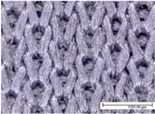	150	0.58 ±0.01	1035 ± 35	1.12	77%	112 ± 5
WO-J	83% PES 12% wool 5% EA	Single jersey	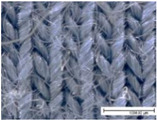	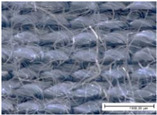	175	0.66 ±0.01/ 0.64 ± 0.01	708 ± 66/ 1033 ± 23	1.36	81%	132 ± 4
HWO-J	46% wool 46% lyocell 8% EA	Single jersey	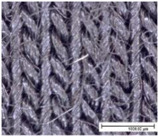	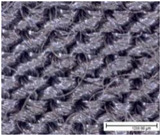	160	0.55 ±0.01 / 0.51 ± 0.01	1917 ± 62/ 1728 ± 36	1.30	78%	113 ± 4
MM-J	95% micromodal 5% EA	Single jersey	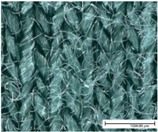	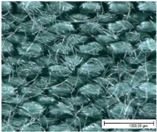	179	0.71 ± 0.01/ 0.68 ± 0.01	1097 ± 22/ 1283 ± 35	1.48	85%	114 ± 3

**Table 3:** Significance levels for the differences between methods at *p* < 0.05 for all evaluated fabrics (* indicates significance at *p* < 0.05).

Fabric/ Method	Maximal Water Content			Drying Time				Drying Rate		
	Two-Sided vs. One-Sided	One-Sided, Unheated vs. Heated	One-Sided, Heated, Unstretched vs. Stretched	Two-Sided, Droplet vs. Fully Wetted	Two-Sided vs. One-Sided	One-Sided, Unheated vs. Heated	One-Sided, Heated, Unstretched vs. Stretched	Two-Sided vs. One-Sided	One-Sided, Unheated vs. Heated	One-Sided, Heated, Unstretched vs. Stretched
PES-I	0.683	0.001 *		<0.001 *	0.001 *	<0.001 *		0.001 *	0.001 *	
PES-J	0.169	<0.001 *	0.347	<0.001 *	<0.001 *	<0.001 *	<0.001 *	0.005 *	<0.001 *	<0.001 *
PES-L1	0.072	0.037 *		<0.001 *	<0.001 *	<0.001 *		0.001 *	<0.001 *	
PES-P	0.002 *	<0.001 *			<0.001 *	<0.001 *		0.009 *	0.013 *	
CO-J	0.894	0.001 *	0.301	<0.001 *	<0.001 *	<0.001 *	0.001 *	0.007 *	<0.001 *	0.002 *
CO-I	0.556	0.007 *			0.001 *	<0.001 *		<0.001 *	<0.001 *	
CO-S	0.049 *	0.031 *		<0.001 *	0.001 *	<0.001 *		<0.001 *	<0.001 *	
PA-I	0.221	0.001 *			<0.001 *	<0.001 *		0.001 *	<0.001 *	
WO-J	0.642	<0.001 *	0.679	<0.001 *	<0.001 *	<0.001 *	0.003 *	0.001 *	<0.001 *	0.001 *
HWO-J	0.841	0.002 *	0.282	0.001 *	0.005 *	<0.001 *	<0.001 *	0.009 *	<0.001 *	0.001 *
MM-J	0.122	<0.001 *	0.029		0.004 *	<0.001 *	0.012 *	<0.001 *	<0.001 *	0.144

**Table 4:** Pearson correlation coefficients for all fabrics and individual fabric groups, such as CO-based, PES-based, and jersey fabrics, and for all methods and parameters used in this study (* means a significance level of <0.05 and ** means *p* < 0.01).

	Maximal Water Content			Drying Time				Drying Rate		
	Two-Sided, Fully Wetted	One-Sided, Fully Wetted	One-Sided, Fully Wetted, Heated	Two-Sided, Droplet Wetted	Two-Sided, Fully Wetted	One-Sided, Fully Wetted	One-Sided, Fully Wetted, Heated	Two-Sided, Fully Wetted	One-Sided, Fully Wetted	One-Sided, Fully Wetted, Heated
All fabrics (11 specimens) vs. mass	0.858 **	0.816 **	0.769 **	0.249	0.814 **	0.839 **	0.948 **	−0.462	−0.621 *	0.192
All fabrics (11 specimens) vs. air permeability	−0.678 *	−0.624 *	−0.599	−0.327	−0.619 *	−0.644 *	−0.701 *	0.550	0.750 **	−0.474
All fabrics (11 specimens) vs. thickness	0.988 **	0.981 **	0.980 **	0.574	0.980 **	0.974 **	0.917 **	−0.578	−0.562	0.504
CO-based fabrics (3 specimens) vs. thickness	0.999 *	0.987	0.999 *	-	0.999 *	0.992	0.990	−0.997 *	−0.997 *	0.999 *
PES-based fabrics (4 specimens) vs. thickness	0.988 *	0.980 *	0.959 *	−0.456	0.992 **	0.997 **	0.919	−0.918	−0.990 **	0.459
Jersey fabrics (5 specimens) vs. thickness	0.551	0.428	0.762	−0.158	−0.261	−0.051	0.127	0.583	0.883 *	0.511
Stretched jersey fabrics (5 specimens) vs. thickness	-	-	0.568	-	-	-	0.790	-	-	−0.322

**Table 5:** Overview of linear correlation coefficients (slopes and intercepts), squared Pearson coefficient (R^2^), and corresponding standard errors (SEs) between fabric thickness and fabric drying characteristics (maximal water content, drying time, and drying rate) for all fabrics and methods used in this study.

Parameter	Maximal Water Content				Drying Time					Drying Rate			
	Unit	Two-Sided, Fully Wetted	One-Sided, Fully Wetted	One-Sided, Fully Wetted, Heated	Unit	Two-Sided, Droplet Wetted	Two-Sided, Fully Wetted	One-Sided, Fully Wetted	One-sided, Fully Wetted, Heated	Unit	Two-Sided, Fully Wetted	One-Sided, Fully Wetted	One-Sided, Fully Wetted, Heated
Slope	(g/m^2^)/mm	1018.9	930.9	797.6	min/mm	46.4	718.9	906.7	66.3	(g/m^2^ min)/mm	−0.444	−0.351	3.515
SE_slope_	(g/m^2^)/mm	53.7	61.8	55.4	min/mm	30.0	49.0	70.7	9.6	(g/m^2^ min)/mm	0.233	0.206	2.019
Intercept	g/m^2^	−119.2	−54.7	−117.1	min	23.8	−132.1	−105.0	2.3	g/m^2^ min	1.918	1.387	5.234
SE_intercept_	g/m^2^	44.1	50.7	45.5	min	24.9	40.2	58.0	7.9	g/m^2^ min	0.191	0.169	1.658
R^2^	-	0.976	0.962	0.958	-	0.324	0.960	0.948	0.842	-	0.288	0.244	0.252
SE	g/m^2^	51.2	59.0	52.9	min	19.1	46.8	67.5	9.1	g/m^2^ min	0.222	0.197	1.928

## Data Availability

The original contributions presented in this study are included in the article. Further inquiries can be directed to the corresponding author.
